# Genome‐wide identification of a novel miRNA‐based signature to predict recurrence in patients with gastric cancer

**DOI:** 10.1002/1878-0261.12385

**Published:** 2018-10-10

**Authors:** Yongmei Yang, Ailin Qu, Rui Zhao, Mengmeng Hua, Xin Zhang, Zhaogang Dong, Guixi Zheng, Hongwei Pan, Hongchun Wang, Xiaoyun Yang, Yi Zhang

**Affiliations:** ^1^ Department of Clinical Laboratory Qilu Hospital Shandong University Jinan China; ^2^ Department of Oral Pathology Institute of Stomatology Qilu Hospital Shandong University Jinan China; ^3^ Department of Gastroenterology Qilu Hospital Shandong University Jinan China

**Keywords:** gastric cancer, miRNA signature, prediction, prognosis, recurrence‐free survival

## Abstract

The current tumor node metastasis (TNM) staging system is inadequate for identifying high‐risk gastric cancer (GC) patients. Using a systematic and comprehensive‐biomarker discovery and validation approach, we attempted to build a microRNA (miRNA)‐recurrence classifier (MRC) to improve the prognostic prediction of GC. We identified 312 differentially expressed miRNAs in 446 GC tissues compared to 45 normal controls by analyzing high‐throughput data from The Cancer Genome Atlas (TCGA). Using a Cox regression model, we developed an 11‐miRNA signature that could successfully discriminate high‐risk patients in the training set (*n* = 372; *P *<* *0.0001). Quantitative real‐time polymerase chain reaction‐based validation in an independent clinical cohort (*n* = 88) of formalin‐fixed paraffin‐embedded clinical GC samples showed that MRC‐derived high‐risk patients succumb to significantly poor recurrence‐free survival in GC patients (*P *<* *0.0001). Cox and stratification analysis indicated that the prognostic value of this signature was independent of clinicopathological risk factors. Time‐dependent receiver operating characteristic (ROC) analysis revealed that the area under the curve of this signature was significantly larger than that of TNM stage in the TCGA (0.733 vs. 0.589 at 3 years, *P *=* *0.004; 0.802 vs. 0.635 at 5 years, *P *=* *0.005) and validation cohort (0.835 vs. 0.689 at 3 years, *P *=* *0.003). A nomogram was constructed for clinical use, which integrated both MRC and clinical‐related variables (depth of invasion, lymph node status and distance metastasis) and did well in the calibration plots. In conclusion, this novel miRNA‐based signature is superior to currently used clinicopathological features for identifying high‐risk GC patients. It can be readily translated into clinical practice with formalin‐fixed paraffin‐embedded specimens for specific decision‐making applications.

**Abbreviations**
AUC: area under receiver operating characteristic curve
CI: confidence interval
FDR: false discover rate
FFPE: formalin‐fixed paraffin‐embedded
GC: gastric cancer
miRNA: microRNA
MRC: miRNA‐recurrence classifier
RFS: recurrence‐free survival
ROC: receiver operating characteristic
TCGA: The Cancer Genome Atlas
TNM: tumor node metastasis

## 1 Introduction

Gastric cancer (GC) is the fourth most common malignancy and ranks as the second leading cause of cancer death worldwide (Siegel *et al*., 2017). Surgical resection with subsequent adjuvant chemoradiotherapy has been considered as a potentially curative treatment for patients with early‐stage GC (Bang *et al*., 2012; Cunningham *et al*., 2006; Macdonald *et al*., 2001; Sasako *et al*., 2011; Stiekema *et al*., 2015). However, recurrence occurs in up to 30–40% of patients within 5 years (Aoyama *et al*., 2011; Bang *et al*., 2012; Lee *et al*., 2012; Sakuramoto *et al*., 2007) and, in turn, 5‐year overall survival estimates range from 5% to 90%, depending largely on the stage of disease at presentation (Aoyama *et al*., 2011; Bang *et al*., 2012; Lee *et al*., 2012; Sakuramoto *et al*., 2007). GC is a clinically heterogeneous disease and it is difficult to accurately predict outcomes even within the same stage. The ability to predict the precise prognosis of an individual patient is critical for the selection of an appropriate treatment plan and follow‐up strategies, whereas the current staging system for GC has shown insufficient prediction for prognosis of patients (Choi *et al*., 2017; Edge and Compton, 2010; Son *et al*., 2012). Hence, the identification of novel markers that could predict survival and relapse in GC would greatly optimize the treatment planning and benefit patients.

Recent advancements in transcriptome profiling have provided compelling evidence of small non‐coding RNA [such as microRNA (miRNA), Piwi‐interacting RNA and small nucleolar RNA] dysregulation in cancers, highlighting the potential of these molecules as diagnostic and prognostic biomarkers (Romano *et al*., 2017). miRNAs, in particular, have shown promising prognostic associations with major cancer outcomes (Nair *et al*., 2012). A growing number of studies have indicated that differentially expressed miRNAs in tumor tissues are key players in oncogenesis and have an impact on the prognosis for GC patients (Kogo *et al*., 2011; Li *et al*., 2015; Nishida *et al*., 2011). Recent findings on the mechanisms of miRNA‐mediated gene regulation in GC also support the development of biomarkers for the precise evaluation of cancer progression (Ishimoto *et al*., 2016). However, because the limited number of miRNAs or patients involved, or different miRNA expression profiling platforms in a GC study, such studies lack a normalized standard.

The Cancer Genome Atlas (TCGA) provides a foundation for systematic analysis of large‐scale miRNA expression data. Most recently, a comprehensive study based on the TCGA and other data platforms has successfully identified an 8‐miRNA signature that significantly predicted recurrence‐free interval in stage II and III colorectal cancer (Kandimalla *et al*., 2018). In the present study, we employed a large cohort of GC patients from the TCGA project and identified a novel miRNA‐based signature for predicting recurrence‐free survival (RFS) in patients with GC, followed by validation of its clinical significance in an independent clinical cohort. Additionally, we assessed the prognostic and predictive value of this signature in the TCGA and validation datasets.

## 2 Materials and methods

### 2.1 Candidate miRNA selection and miRNA signature identification using TCGA data

Data for selected samples of 446 GC patients and 45 normal controls were downloaded from The Cancer Genome Atlas Cancer Genome (https://portal.gdc.cancer.gov). The dataset acquired above contained 1881 noted miRNA expression data. The downloaded clinicopathological information and follow‐up data were matched with the miRNA expression profiles. The RFS events included the first recurrence of GC at a local, regional or distant site, and death from any cause. Patients without events or death were censored at the time of last follow‐up.

The TCGA GC patients were used as the training cohort for identifying prognostic miRNAs and building the miRNA‐recurrence classifier (MRC). First, TCGA miRNA data were log2 transformed and the miRNA expression levels between non‐cancer and cancer were compared using the criteria: absolute log2 fold‐change > 1, false discovery rate (FDR) < 0.05 and relatively high expression levels of miRNAs (count per million > 1). Subsequently, differentially expressed miRNAs were subjected to univariate Cox proportional hazards regression analysis. The miRNAs with *P* < 0.05 were considered as the candidate prognostic miRNAs of RFS and entered into multivariate Cox proportional hazards regression. To identify the independent predictors that significantly contributed to RFS, we used the least value of Akaike information criterion (AIC) with respect to miRNA selection and the established MRC. The risk score of each patient was calculated to predict RFS of GC, with the regression coefficients of multivariate Cox regression model being used to weight each miRNA expression level in the prognostic classifier:

$$\text{Risk}\;\text{Score}=\sum_{i}\text{coefficient}\;\left({\text{miRNA}}_{i}\right)\times \text{expression}\;\left({\text{miRNA}}_{i}\right)$$

Using the optimum cut‐off value obtained from x‐tile plots (x‐tile, version 3.6.1; Yale University School of Medicine, New Haven, CT, USA), patients were categorized into high‐risk and low‐risk groups.

### 2.2 Patient and sample collection

In total, 88 formalin‐fixed paraffin‐embedded (FFPE) specimens were collected from GCs patients who underwent radical surgery at Qilu Hospital, Shandong University between 2012 and 2014. All samples were evaluated by two pathologists in accordance with the American Joint Committee on Cancer TNM grading system (7th edition) (https://cancerstaging.org). All procedures performed in the study involving human participants were conducted in accordance with the ethical standards of the Clinical Research Ethics Committee of Qilu Hospital, Shandong University and the Declaration of Helsinki. The experiments were undertaken with the understanding and written consent of each subject.

### 2.3 RNA isolation, cDNA synthesis and quantitative real‐time polymerase chain reaction

Total RNA extraction from 10‐μm thick FFPE specimens was performed using miRNA isolation Kits (Bioteke, Beijing, China). All RNA manipulations were carried out under RNase‐free conditions and cDNA was synthesized using miRNA‐specific Bugle‐Loop primers (Ribobio, Guangzhou, China) and the M‐MLV RT kit in accordance with the manufacturer's recommendations (Invitrogen, Carlsbad, CA, USA). miRNA expression was assessed by a quantitative real‐time polymerase chain reaction using ABI PRISM 7500 Sequence Detection System (Applied Biosystems, Foster City, CA, USA). The relative levels of miRNA expression were determined using the $2^{-\Delta C_{T}}$ method with the U6 small nuclear RNA (U6) as the reference gene to normalize the data. The normalized values were further log2 transformed. All primers for miRNAs used in this part of the study were purchased from Ribobio.

### 2.4 Statistical analysis

prism, version 7.0 (GraphPad Software Inc., San Diego, CA, USA) and r, version 3.4.0 (http://www.Rproject.org) were used to analyze all the data. *P *<* *0.05 was considered statistically significant. Differential expression analysis of miRNAs between non‐cancer and cancer groups was performed using the edgeR package of r (Robinson *et al*., 2010). In survival analyses, we used the Kaplan–Meier method to draw survival curves, which were compared by log‐rank tests. A Cox proportional hazard regression model was applied for the univariate analysis and multivariate analysis of prognostic factors. The prognostic or predictive accuracy of each variable was investigated using time‐dependent receiver operating characteristic (ROC) analysis in the survivalROC package and the bootstrap method was applied to test the significance of differences between the ROC curves. The regression coefficients in multivariable Cox regression model were used to generate the nomogram. A calibration plot was used to explore the agreement of nomogram between predictions and observations. Nomogram and calibration plot were performed using the rms package of r software.

## 3 Results

### 3.1 Identification of GC‐specific miRNAs by analyzing the TCGA dataset

Based on the miRNA expression data from the TCGA dataset, we compared miRNA expression profiles between 446 GC and normal 45 control groups and found 312 miRNAs with an absolute fold‐change differences of 2 and a FDR < 0.05 (Table S1). These significantly differentially expressed miRNAs were considered as candidate prognostic biomarkers for GC patients, among which 260 miRNAs were identified as upregulated and 52 as downregulated in GC compared to normal control (Fig. S1).

### 3.2 Identification of the prognostic miRNAs from the training cohort

To single out the prognostic miRNAs, 312 GC‐specific miRNAs were initially subjected to univariate Cox proportional hazards regression analysis in 372 patients for whom complete miRNA data, clinicopathological characteristics and follow‐up information were available. In total, 24 miRNAs were found to be significantly associated with the GC patient RFS (*P* < 0.05) (Table S2) and were subsequently entered into a multivariate Cox regression analysis. For the purpose of identifying the best predictors that significantly contributed to patient RFS, we used the lowest value of the Akaike information criterion for variable selection and built a prognostic classifier, which consisted of 11 miRNAs (miR‐365a, miR‐145, miR‐181b, miR‐549a, miR‐708, miR‐7‐3, miR‐378i, miR‐466, miR‐3923, miR‐4793 and miR‐3144). Among these miRNAs, seven (miR‐145, miR‐549a, miR‐7‐3, miR‐378i, miR‐466, miR‐4793 and miR‐3144) with a negative coefficient were protective factors as a result of the close association between their high expression and a longer patient RFS, whereas the remaining four (miR‐365a, miR‐181b, miR‐708 and miR‐3923) were risk factors.

### 3.3 Construction of a miRNA prognostic risk model and its predictability assessment in the training cohort

**Figure 1:**
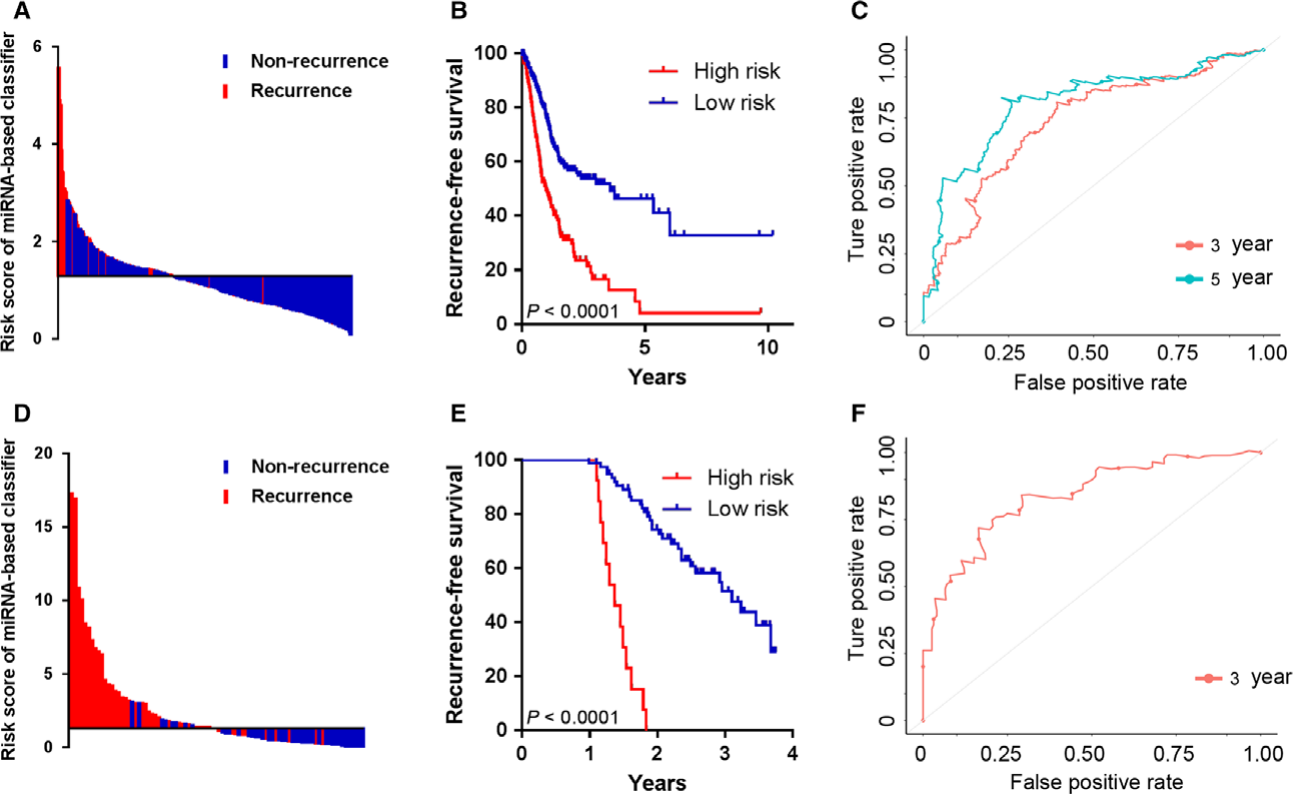
Risk score by the 11‐miRNA classifier, time‐dependent ROC curves and Kaplan–Meier survival in the training set (A–C) and validation set (D–F).

Using the regression coefficients of multivariate Cox regression model to weight each miRNA expression level in the MRC, we developed a risk score formula to predict patient survival: risk score = miR‐365a × 0.15853 +  miR‐145 × (−0.14044) + miR‐181b × 0.22993 + miR‐549a × (−0.15682) + miR‐708 × 0.13047 + miR‐7‐3 × (−0.1562) + miR‐378i × (−0.37045) + miR‐466 × (−0.23334) + miR‐3923 × 0.25761 + miR‐4793 × (−0.6004 6) + miR‐3144 × (−0.25687). We then calculated the risk scores for all GC patients using this formula. Using x‐tile plots to generate the optimum cut‐off value (Fig. S2A), we included those patients with a risk score of 1.30 or lower in the group of patients at low risk of disease recurrence (low‐risk group) and also those with a risk score higher than 1.30 in the high‐risk group. Patients with a higher risk score generally had poorer survival than those with lower risk score and the distribution of risk scores and survival status is shown in Fig. 1A. Kaplan–Meier survival analysis demonstrated that patients with high‐risk scores had a shorter RFS than those with low‐risk scores (log‐rank test, *P *<* *0.001) (Fig. 1B). Figure 1C shows the predictive potential of MRC using time‐dependent ROC curves. The area under the ROC curve (AUC) of the prognostic model for RFS was 0.733 at 3 years and 0.802 at 5 years. In the univariate Cox regression model of RFS, the high‐risk group showed a 2.492‐fold increased risk of recurrence [95% confidence interval (CI) 1.867–3.326, *P *=* *5.78 × 10^–10^] compared to the low‐risk group (Table 1).

**Table 1:** Variables associated with RFS according to the Cox proportional hazards regression model

Variable	Univariable analysis			Multivariable analysis		
	Hazard ratio	95% CI	*P* value	Hazard ratio	95% CI	*P* value
Age	1.01	0.997–1.024	0.148			
Sex						
Male vs. female	1.272	0.935–1.731	0.126			
*Helicobacter pylori* infection						
Yes vs. no	0.435	0.175–1.079	0.072			
Histologic grade						
G1	Ref	–	–			
G2	0.5742	0.180–1.838	0.350			
G3	0.8426	0.268–2.652	0.770			
Stage						
Stage I	Ref	–	–			
Stage II	1.383	0.784–2.441	0.263			
Stage III	2.005	1.180–3.408	0.010			
Stage IV	3.447	1.839–6.462	0.000			
T						
T1	Ref	–	–	Ref	–	–
T2	4.014	1.240–13.00	0.020	2.869	0.880–9.349	0.080
T3	4.173	1.322–13.18	0.015	2.378	0.740–7.648	0.146
T4	4.055	1.265–13.00	0.018	2.107	0.643–6.900	0.218
N						
N0	Ref	–	–	Ref	–	–
N1	1.86	1.237–2.797	0.003	1.593	1.049–2.421	0.029
N2	1.739	1.115–2.711	0.015	1.586	1.009–2.494	0.046
N3	2.676	1.769–4.047	0.000	2.114	1.371–3.261	0.001
M						
M1 vs. M0	1.968	1.158–3.344	0.012	2.048	1.174–3.573	0.012
MRC						
High vs. low	2.492	1.867–3.326	0.000	2.327	1.731–3.129	0.000

### 3.4 Validation of the miRNA classifier for RFS prediction in the validation cohort

To determine whether the MRC derived from the TCGA cohort was robust, we measured its performance in an independent validation cohort, which comprised 88 FFPE tissues from GC patients. We examined the expression levels of all 11 miRNAs in GC tissues and constructed a prognostic classifier using a Cox proportional hazard model. Based upon the Cox‐model derived risk scores, patients in the validation cohort were dichotomized into low and high‐risk groups, using the x‐tile derived cut‐off threshold (Fig. S2B). In line with the results from the TCGA cohort, more patients with recurrence fell into the high‐risk group (Fig. 1D), in which the RFS was shorter than that in low‐risk group (Fig. 1E). Furthermore, the MRC achieved an AUC of 0.835, which was clinically interesting (Fig. 1F).

### 3.5 Prognostic value of the miRNA classifier

**Figure 2:**
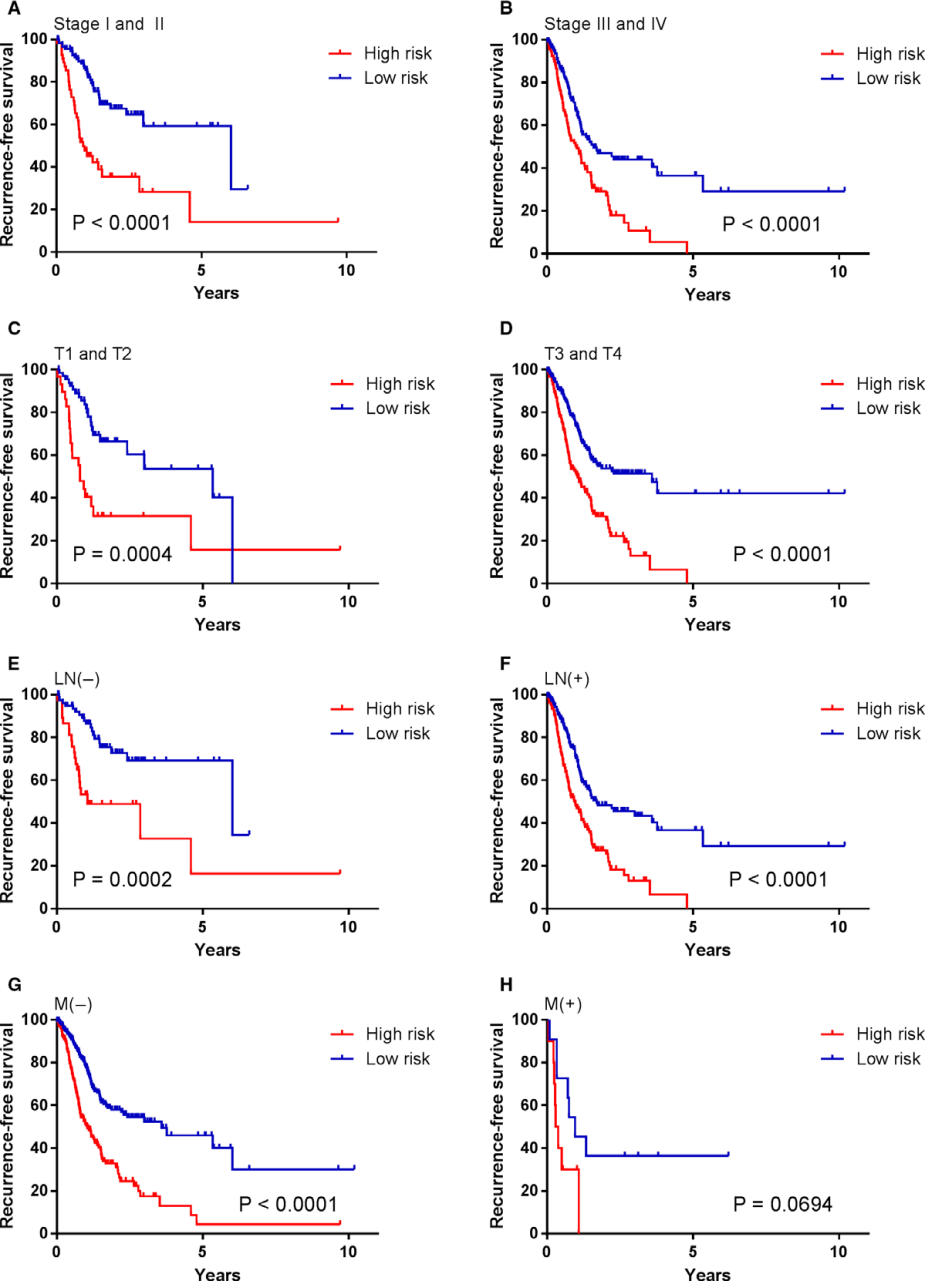
Kaplan–Meier survival analysis according to the 11‐miRNA classifier stratified by clinicopathological risk factors in the TCGA cohort. (A, B) TNM stage. (C, D) T stage. (E, F) lymph node status. (G, H) M stage. *P* values were calculated using the log‐rank test.

**Figure 3:**
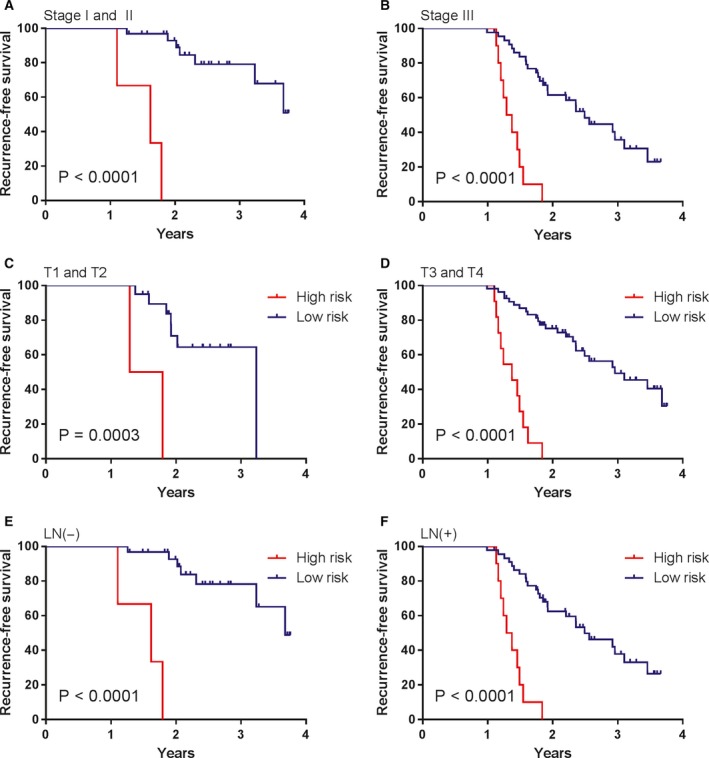
Kaplan–Meier survival analysis according to the 11‐miRNA classifier stratified by clinicopathological risk factors in the validation cohort. (A, B) TNM stage. (C, D) T stage. (E, F) lymph node status.

**Figure 4:**
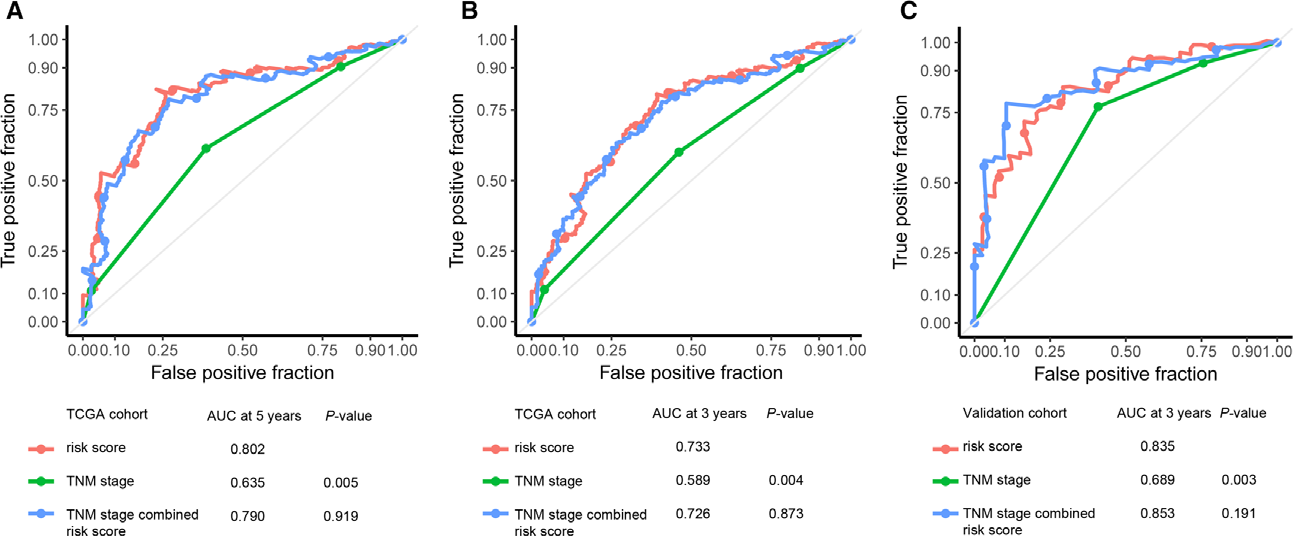
Time‐dependent ROC curves to compare the prognostic accuracy of the 11‐miRNA classifier with tumor stage in the training cohort (A, B) and validation cohort (C).

To investigate whether the prognostic value of MRC was independent of other clinicopathological variables, the univariable and multivariable Cox regression analyses were initially performed in the TCGA cohort. We found that risk score was significantly associated with RFS even when adjusted by other clinical factors (Table 1). We also observed that clinical stages and clinicopathologic classifications (T, N and M) were significant in Cox regression analyses. Therefore, stratification analysis was introduced to determine the independence of MRC according to clinical stages and T, N and M classifications. As shown in Fig. 2, the high‐risk survival curves were below the low‐risk curves in all subgroups, including TNM stage (Stage I and II, Fig. 2A; Stage III and IV, Fig. 2B); T stage (T1 and T2, Fig. 2C; T3 and T4, Fig. 2D); lymph node status (LN−, Fig. 2E; LN+, Fig. 2F); and M stage (M−, Fig. 2G; M+, Fig. 2H). A log‐rank test showed that MRC was still a clinically and statistically significant prognostic signature in all subgroups except for distant metastasis group (M+ group). For the M+ subgroup, the difference was marginal (*P *=* *0.0694). This was probably because the sample size was too small (only 21 patients) to draw any firm conclusions. Stratification analysis yielded similar results in the validation cohort (Fig. 3). We also performed ROC analysis to compare the prognostic accuracy of MRC with tumor stage. Figure 4A,B shows that the 11‐miRNA risk score model possessed a stronger predictive power than TNM stage for the prognostic evaluation of GC patients in the TCGA cohort (0.733 vs. 0.589, 95% CI = 0.613–0.853 vs. 0.514–0.664 at 3 years; 0.802 vs. 0.635, 95% CI = 0.652–0.952 vs. 0.548–0.722 at 5 years; *P *=* *0.005). When the MRC was combined with the TNM stage, no significant difference was found between the combined model and the MRC (*P *>* *0.05). Subsequent analysis in the FFPE tissues produced similar results (Fig. 4C). The results from the validation dataset further confirmed the reliable predictive ability of MRC.

### 3.6 Construction of nomogram based on the miRNA classifier

**Figure 5:**
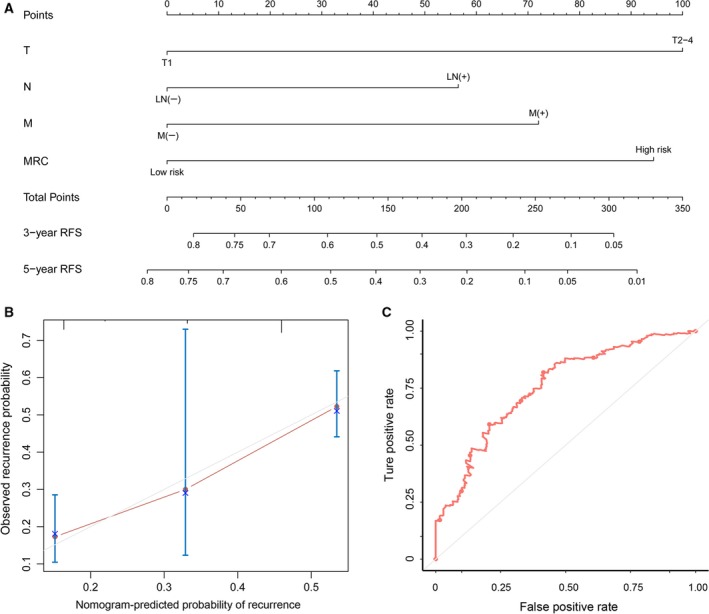
The nomogram to predict probability of RFS for CRC patients in the training set. (A) The nomogram for predicting the proportion of patients with RFS. (B) The calibration plot of the nomogram for the probability of RFS at 3 years. (C) Time‐dependent ROC based on the nomogram for recurrence probability. AUC = 0.754. Nomogram‐predicted probability of recurrence is plotted on the *x*‐axis and observed recurrence is plotted on the *y*‐axis. The red line represents our nomogram and the vertical bars represent the 95% CI.

To provide the clinician with a quantitative method for predicting the individual probability of cancer recurrence, we built a nomogram that integrated the MRC and clinicopathological independent risk factors for RFS (Fig. 5A). The bias‐corrected line in the calibration plot was found to be closer to the ideal curve (the 45° line), which indicated good agreement between prediction and observation (Fig. 5B). The predictive accuracy of the nomogram was calculated via ROC analysis: the AUC of nomogram was 0.754, which implied that the discrimination performance was favorable (Fig. 5C).

## 4 Discussion

An effective molecular‐based method for predicting prognosis in cancer patients is urgently needed to optimize tailored treatment in the era of precision medicine. In the present study, we identified an 11‐miRNA signature that is associated with tumor recurrence in GC patients using high‐throughput data from the TCGA database. We confirmed these findings in an independent clinical cohort of GC patients. Patients with a high‐risk score for this 11‐miRNA signature had increased tumor recurrence, even after stratifying patients by clinicopathological risk factors.

Previous studies on miRNA expression profiling have consistently revealed that the miRNA‐based signature is a potential predictor for progression or relapse in patients with various types of cancers, including GC (Li *et al*., 2010; Ueda *et al*., 2010). Moreover, the TCGA has profiled and analyzed large numbers of human cancers to identify molecular aberrations at the DNA, RNA, proteomic and epigenetic levels (Weinstein *et al*., 2013). Recently, Sohn *et al*. (2017) developed a model based on four molecular subtypes of GC from the TCGA project to predict survival and adjuvant chemotherapy outcomes. However, to date, the miRNA expression signatures from the TCGA database with respect to the survival prognosis of GC have not been investigated systematically. The miRNA predictive signature for survival meets some crucial standards: (i) its expression must be specific in cancer and non‐cancer; (ii) it is correlated with patient survival; and (iii) it has a synergized effect in the survival prognosis. Based on the genome‐wide discovery of GC‐specific miRNAs in the TCGA dataset, we identified 11 miRNAs that were strongly related to RFS in GC patients. In agreement with our findings, similar studies have already been carried out for the identification of prognostic miRNA signatures from the TCGA in other types of cancers, including bladder cancer, glioblastoma, colon cancer, etc. (Gonzalez‐Vallinas *et al*., 2018; Hermansen *et al*., 2017; Liu *et al*., 2017a; Wong *et al*., 2016; Xu *et al*., 2016; Zhou *et al*., 2015).

Among the identified miRNAs in the present study, we found that four miRNAs, including miR‐365a, miR‐181b‐1, miR‐708 and miR‐3923, were risk factors, whereas the other seven, including miR‐145, miR‐549a, miR‐7‐3, miR‐378i, miR‐466, miR‐4793 and miR‐3144, were protective factors. High levels of risk factors and low levels of protective factors were independent negative prognostic factors according to our multivariate analysis. These results are consistent with previous research showing that miR‐181b was involved in transforming growth factor beta‐induced epithelial‐to‐mesenchymal transition and GC metastasis (Zhou *et al*., 2016). Considering protective factors, we noted that miR‐145 could inhibit the malignant phenotypes and suppress metastasis of GC via different molecular mechanisms (Gao *et al*., 2013; Lei *et al*., 2017; Tong *et al*., 2018; Xing *et al*., 2015; Xue *et al*., 2016; Zheng *et al*., 2013). In another study, miR‐7‐3 was confirmed to be an independent prognostic indicator for stomach adenocarcinoma (Huo, 2017). Two miRNAs, miR‐466 and miR‐3144, have been indicated as tumor suppressors in other types of cancers (Colden *et al*., 2017; Lin *et al*., 2015; Tong *et al*., 2018), although there are no data available regarding their roles in GC. Other miRNAs, miR‐365a, miR‐3923, miR‐549a, miR‐378i and miR‐4793, are also reported for the first time in GC in the present study. Although it appears that miR‐708 as a risk factor was inconsistent with previous studies reporting its anti‐oncogenic role in GC (Li *et al*., 2018), increased miR‐708 expression and its association with poor survival in lung adenocarcinoma has been demonstrated by other researchers (Jang *et al*., 2012). Thus, further studies are required to comprehensively assess the exact contribution of these miRNAs in tumor progression.

The combined analysis of a panel of multiple factors, rather than a single biomarker, will have more power to provide clinically useful information. An ideal prognostic classifier for GC risk prediction should be robust and potentially feasible in FFPE samples that would overcome barriers of sample collection and storage. A validation from FFPE samples was performed and the combined index of the 11 miRNAs showed a significant association with survival in GC patients. The results of multivariate analysis showed that the 11‐miRNA signature is independent of traditional clinical risk factors. When the stratification analysis was performed, we found the 11‐miRNA signature could discriminate patients at high‐risk from those at low‐risk within all subgroups. Time‐independent ROC analysis showed that our 11‐miRNA signature was superior to TNM stage for prognostic evaluation. To improve the ability of prognostic prediction, we combined the 11‐miRNA signature with TNM stage. However, there was no significant difference between the combined model and our miRNA signature, indicating that our 11‐miRNA signature could yield reliable predictive ability by itself.

It has been reported that miRNAs are sufficiently stable to be detected in both FFPE and blood samples (Hall *et al*., 2012; Mitchell *et al*., 2008). Circulating miRNAs might enable successful close monitoring for early signs of cancer relapse because serum molecules are easily detectable and are also acceptable for patients. Previously, a 7‐miRNA classifier within plasma was reported to predict tumor recurrence in stage II and III GC (Liu *et al*., 2017b); however, our miRNA classifier illustrated its ability to predict recurrence in all stages of GC and performed well. Although we did not have access to blood specimens, it is very likely that our miRNA signatures may eventually be translated into a blood‐based surveillance assay.

Prognostic nomograms comprise the visualization of statistical models specifically developed to optimize the predictive accuracy of individuals, enabling a more individualized prediction of outcome based on a combination of variables (Balachandran *et al*., 2015; Iasonos *et al*., 2008; Shariat *et al*., 2008). Regarding GC, some models based on clinical‐associated factors, such as age, tumor size, tumor invasion depth and lymph node involvement, were demonstrated to be useful for prognosis prediction in patients with GC (Hirabayashi *et al*., 2014; Kim *et al*., 2015). However, an optimal approach that combines multiple miRNA biomarkers and clinical risk factors as a predictive model has yet to be developed. In the present study, we built a nomogram that integrated the miRNA‐based classifier and clinicopathological independent risk factors for RFS. The nomogram performed well in the calibration plot, which indicated good agreement between prediction and observation. The ROC for the prediction nomogram was 0.754, which implied that the discrimination performance was favorable. Therefore, our nomogram may be an important tool for risk stratification and prognosis prediction in GC patients, aiding in individualized treatment decisions and postoperative counseling, and ultimately contributing to improved survival.

## 5 Conclusions

In conclusion, the present study revealed a novel, robust 11‐miRNA classifier for tumor recurrence prediction in patients with GC. This approach can be readily deployed in clinical practice with FFPE samples and achieved superior predictive accuracy compared to currently used clinicopathological risk factors. Moreover, the 11‐miRNA classifier was an independent prognostic factor for, and had a better prognostic ability than, clinical risk factors. Also, these findings should be validated in large‐scale multicenter clinical trials.

## Author contributions

YZ conceived and designed the experiments. YY, AQ, XZ and ZD performed all of the experiments. YY, AQ, RZ, GZ, HP, HW and XY analyzed the data. YY and AQ wrote the manuscript. MH provided the pathological samples. All authors read and approved the final manuscript submitted for publication.

## Supporting information


**Fig. S1.** Volcano plot showing the differentially expressed miRNAs between gastric tumors and normal tissue samples from the TCGA cohort.
**Fig. S2.** x‐tile plots of the miRNA‐recurrence classifier and the risk score in the training (A) and validation (B) sets.
**Table S1.** Summary of 312 differentially expressed microRNAs.
**Table S2.** Identification of the prognostic miRNAs from the TCGA cohort.
