# Supplementary material for: Genome‐wide identification of a novel miRNA‐based signature to predict recurrence in patients with gastric cancer
Source: Mol Oncol. 2018 Oct 10;12(12):2072–84. doi: 10.1002/1878-0261.12385 (PMC6275280; doi:10.1002/1878-0261.12385)
Supplement: Supplementary file 1 — Fig. S1. Volcano plot showing the differentially expressed miRNAs between gastric tumors and normal tissue samples from the TCGA cohort. Fig. S2. x‐tile plots of the miRNA‐recurrence classifier and the risk score in the training (A) and validation (B) sets. Table S1. Summary of 312 differentially expressed microRNAs. Table S2. Identification of the prognostic miRNAs from the TCGA cohort. [file MOL2-12-2072-s001.pdf]

Supplementary Figure 1. Volcano plot showing the differentially expressed miRNAs between gastric tumor and normal tissue samples from the TCGA cohort.

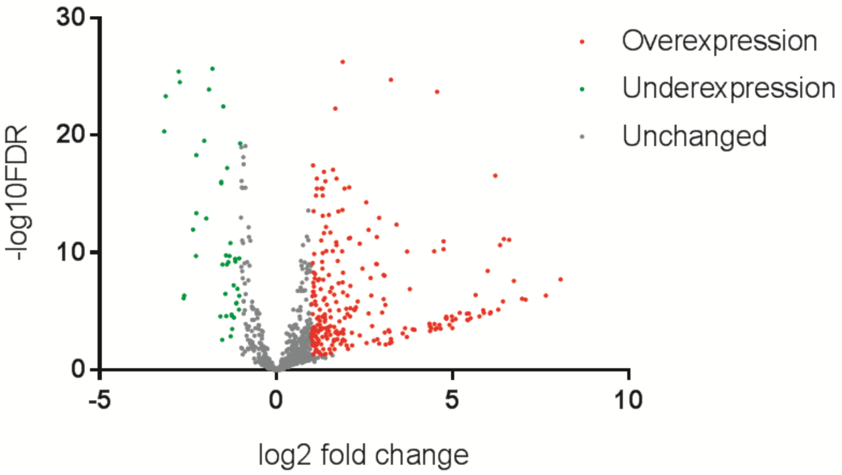

Supplementary Figure 2.

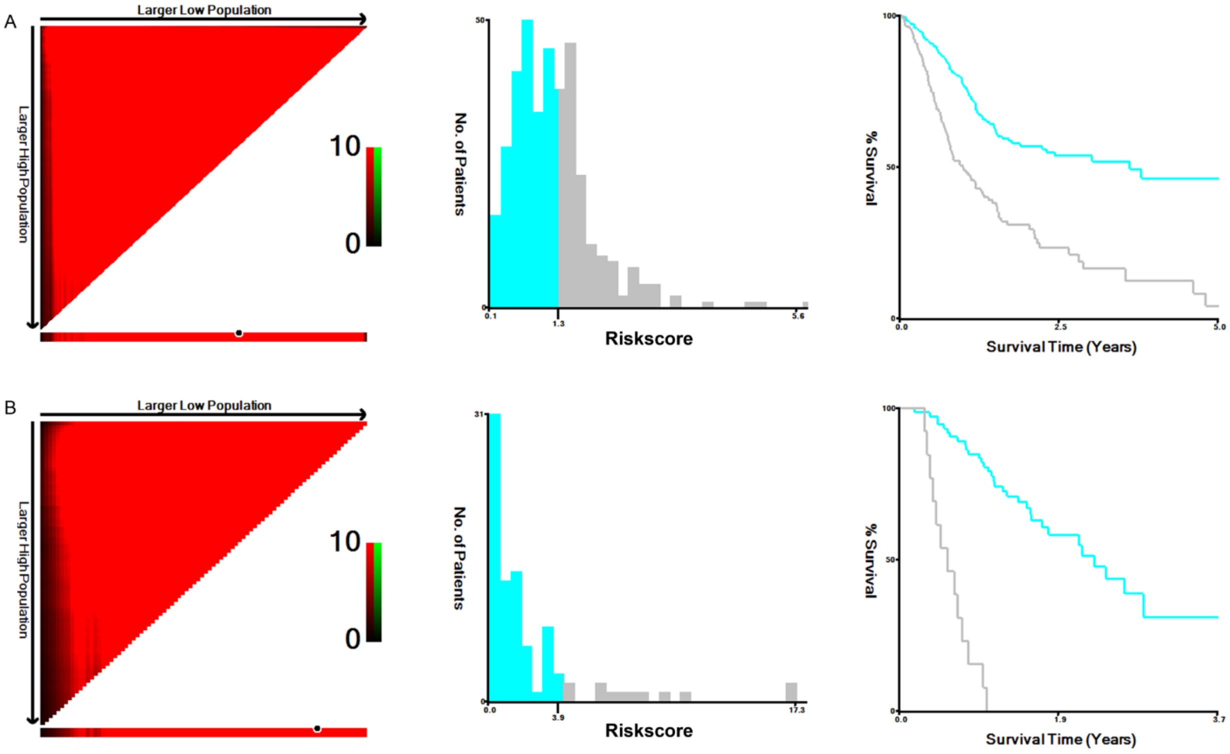

Supplementary Table 1. Summary of 312 differentially expressed microRNAs.

gene	logFC	PValue	FDR
hsa-mir-6510	-4.307	2.09E-37	5.33E-35
hsa-mir-490	-3.167	1.26E-22	4.61E-21
hsa-mir-6507	-3.127	1.07E-25	4.53E-24
hsa-mir-133a-2	-3.010	2.48E-35	2.99E-33
hsa-mir-133a-1	-2.976	3.93E-35	4.14E-33
hsa-mir-1-1	-2.896	2.32E-33	1.96E-31
hsa-mir-1-2	-2.884	4.40E-33	3.37E-31
hsa-mir-5683	-2.765	6.54E-28	3.67E-26
hsa-mir-133b	-2.732	5.75E-27	2.85E-25
hsa-mir-205	-2.618	1.65E-07	7.64E-07
hsa-mir-6499	-2.600	9.94E-08	4.71E-07
hsa-mir-548ba	-2.353	8.29E-14	1.06E-12
hsa-mir-944	-2.270	2.28E-11	1.96E-10
hsa-mir-885	-2.262	1.64E-20	4.93E-19
hsa-mir-383	-2.259	3.00E-15	4.44E-14
hsa-mir-145	-2.237	9.73E-37	1.37E-34
hsa-mir-139	-2.106	2.17E-49	9.16E-47
hsa-mir-551b	-2.041	8.19E-22	2.88E-20
hsa-mir-204	-1.979	9.06E-15	1.23E-13
hsa-mir-143	-1.901	2.59E-26	1.21E-24
hsa-mir-29c	-1.805	3.38E-28	2.03E-26
hsa-mir-137	-1.578	8.11E-06	2.78E-05
hsa-mir-486-1	-1.553	5.84E-18	1.20E-16
hsa-mir-486-2	-1.551	4.31E-18	9.09E-17
hsa-mir-802	-1.525	0.001329908	0.002761361
hsa-mir-1258	-1.522	1.43E-10	1.05E-09
hsa-mir-195	-1.492	8.38E-25	3.37E-23
hsa-mir-202	-1.435	6.68E-08	3.31E-07
hsa-mir-6720	-1.420	1.93E-11	1.68E-10
hsa-mir-378i	-1.411	7.40E-06	2.56E-05
hsa-mir-203a	-1.395	1.43E-10	1.05E-09
hsa-mir-30a	-1.388	2.36E-19	6.21E-18
hsa-mir-365a	-1.366	3.05E-37	5.33E-35
hsa-mir-365b	-1.365	3.16E-37	5.33E-35
hsa-mir-605	-1.353	8.17E-11	6.44E-10
hsa-mir-451a	-1.323	2.30E-11	1.96E-10
hsa-mir-149	-1.294	1.53E-12	1.54E-11
hsa-mir-4501	-1.285	0.0006351	0.00140892
hsa-mir-129-2	-1.264	5.50E-06	1.92E-05
hsa-mir-129-1	-1.257	6.99E-06	2.42E-05
hsa-mir-187	-1.249	0.000118719	0.000316709

hsa-mir-100	-1.200	1.10E-08	6.07E-08
hsa-mir-4732	-1.192	1.05E-05	3.49E-05
hsa-mir-218-2	-1.160	4.23E-11	3.53E-10
hsa-mir-218-1	-1.157	6.98E-11	5.66E-10
hsa-mir-504	-1.131	5.05E-07	2.15E-06
hsa-let-7c	-1.118	4.59E-07	1.97E-06
hsa-mir-144	-1.092	2.29E-08	1.22E-07
hsa-mir-153-1	-1.054	1.85E-06	7.28E-06
hsa-mir-378d-2	-1.045	1.01E-07	4.74E-07
hsa-mir-6511b-2	-1.045	3.58E-11	3.02E-10
hsa-mir-28	-1.019	1.41E-21	4.75E-20
hsa-mir-3942	1.004	0.000759049	0.001657715
hsa-mir-3194	1.008	0.000341938	0.000803161
hsa-mir-141	1.016	5.88E-07	2.49E-06
hsa-mir-203b	1.024	0.000396434	0.000918116
hsa-mir-200c	1.026	1.12E-07	5.26E-07
hsa-mir-548s	1.026	0.000210915	0.000519887
hsa-mir-6805	1.029	0.00438711	0.007987762
hsa-mir-4640	1.034	0.001615035	0.003280661
hsa-mir-362	1.038	9.77E-11	7.56E-10
hsa-mir-95	1.039	4.89E-06	1.73E-05
hsa-mir-106b	1.044	1.33E-19	3.63E-18
hsa-mir-3652	1.044	0.000138552	0.000360492
hsa-mir-34c	1.047	4.39E-08	2.21E-07
hsa-mir-3691	1.049	0.000831899	0.00180745
hsa-mir-550a-2	1.054	6.66E-09	3.74E-08
hsa-mir-4660	1.054	0.000299878	0.000712104
hsa-mir-3144	1.059	0.029260734	0.044930416
hsa-mir-532	1.060	1.85E-15	2.84E-14
hsa-mir-320b-1	1.061	4.41E-08	2.21E-07
hsa-mir-130b	1.062	7.55E-10	4.93E-09
hsa-mir-320b-2	1.067	1.41E-08	7.65E-08
hsa-mir-3177	1.073	0.003587419	0.006646581
hsa-mir-6777	1.075	0.003402985	0.006346718
hsa-mir-4726	1.076	0.002673458	0.005169095
hsa-mir-548o	1.077	7.65E-05	0.000218571
hsa-mir-3117	1.080	0.000191566	0.000480625
hsa-mir-500b	1.083	1.47E-11	1.29E-10
hsa-mir-559	1.092	0.000565461	0.001277972
hsa-mir-3934	1.098	2.53E-09	1.54E-08
hsa-mir-4766	1.101	0.000104696	0.000283791
hsa-mir-3677	1.101	9.86E-08	4.71E-07
hsa-mir-421	1.104	9.99E-10	6.33E-09
hsa-mir-5580	1.105	0.005496048	0.009795282

hsa-mir-4797	1.105	0.000105379	0.000284725
hsa-mir-5703	1.106	0.000166871	0.000423711
hsa-mir-223	1.108	3.36E-07	1.48E-06
hsa-mir-588	1.113	0.015996973	0.025784796
hsa-mir-7112	1.123	0.000570506	0.001285927
hsa-mir-3682	1.130	6.90E-07	2.86E-06
hsa-mir-1307	1.130	8.52E-17	1.41E-15
hsa-mir-940	1.130	3.70E-07	1.61E-06
hsa-mir-4793	1.139	0.010453871	0.017450719
hsa-mir-4677	1.152	1.97E-17	3.39E-16
hsa-mir-181a-1	1.156	2.07E-18	4.85E-17
hsa-mir-5582	1.160	0.00095966	0.002074341
hsa-mir-6850	1.161	0.017409177	0.027795334
hsa-mir-6728	1.163	0.003711484	0.006846349
hsa-mir-7854	1.166	1.25E-05	4.03E-05
hsa-mir-3680-1	1.174	0.000197458	0.000490624
hsa-mir-1229	1.177	4.86E-05	0.000144443
hsa-mir-5586	1.179	5.14E-09	2.95E-08
hsa-mir-3145	1.181	0.002367561	0.004609362
hsa-mir-200b	1.186	3.91E-09	2.27E-08
hsa-mir-548k	1.187	0.000254496	0.000618271
hsa-mir-4645	1.194	1.25E-05	4.03E-05
hsa-mir-3200	1.203	9.23E-06	3.07E-05
hsa-mir-6718	1.204	0.0014949	0.003081175
hsa-mir-4431	1.213	0.017730541	0.028254907
hsa-mir-643	1.222	3.09E-09	1.85E-08
hsa-mir-4461	1.235	4.87E-05	0.000144443
hsa-mir-3684	1.236	2.06E-05	6.38E-05
hsa-mir-548v	1.241	1.80E-06	7.15E-06
hsa-mir-216a	1.242	0.000153395	0.000391855
hsa-mir-3196	1.243	0.026031608	0.040488275
hsa-mir-194-1	1.263	2.41E-08	1.27E-07
hsa-mir-4479	1.263	9.56E-05	0.000266867
hsa-mir-492	1.269	0.031895356	0.048621673
hsa-mir-181b-1	1.275	1.93E-17	3.39E-16
hsa-mir-7844	1.280	0.00014534	0.000374684
hsa-mir-6844	1.281	0.003450564	0.006421249
hsa-mir-550a-1	1.283	8.38E-13	8.94E-12
hsa-mir-708	1.304	7.43E-11	5.97E-10
hsa-mir-200a	1.312	3.32E-10	2.29E-09
hsa-mir-3150b	1.313	0.000684759	0.001507185
hsa-mir-210	1.313	8.42E-06	2.84E-05
hsa-mir-181b-2	1.319	8.14E-17	1.37E-15
hsa-mir-1301	1.319	5.22E-15	7.46E-14

hsa-mir-3619	1.320	9.74E-05	0.000269156
hsa-mir-93	1.320	1.87E-17	3.35E-16
hsa-mir-503	1.324	1.23E-11	1.09E-10
hsa-mir-194-2	1.334	3.45E-09	2.04E-08
hsa-mir-3660	1.336	0.01060955	0.017640731
hsa-mir-7-2	1.347	2.47E-07	1.11E-06
hsa-mir-500a	1.351	5.34E-19	1.32E-17
hsa-mir-3127	1.355	1.77E-13	2.16E-12
hsa-mir-618	1.375	2.51E-05	7.69E-05
hsa-mir-3174	1.376	8.72E-06	2.92E-05
hsa-mir-4326	1.380	1.56E-07	7.24E-07
hsa-mir-5698	1.383	5.11E-06	1.79E-05
hsa-mir-3689b	1.385	0.029550791	0.045211102
hsa-mir-7702	1.389	1.58E-05	5.01E-05
hsa-mir-17	1.393	3.86E-18	8.34E-17
hsa-mir-3689a	1.403	0.028994827	0.044603356
hsa-mir-3618	1.410	0.000354788	0.000828495
hsa-mir-20a	1.411	4.93E-14	6.49E-13
hsa-mir-5092	1.411	0.00010129	0.000277232
hsa-mir-4746	1.418	7.78E-12	7.24E-11
hsa-mir-4684	1.422	0.000620349	0.001379825
hsa-mir-1254-1	1.438	6.64E-07	2.78E-06
hsa-mir-548d-2	1.440	0.000136653	0.00035776
hsa-mir-3131	1.450	0.001042266	0.002230027
hsa-mir-548d-1	1.476	8.14E-05	0.000231035
hsa-mir-188	1.492	4.29E-15	6.23E-14
hsa-mir-4741	1.493	0.000198864	0.000491621
hsa-mir-1292	1.496	1.28E-09	8.03E-09
hsa-mir-548x	1.511	0.010732323	0.017739899
hsa-mir-6783	1.522	2.35E-06	8.90E-06
hsa-mir-7-3	1.526	4.31E-08	2.19E-07
hsa-mir-6821	1.535	0.000455819	0.001047017
hsa-mir-4449	1.538	6.14E-05	0.000179221
hsa-mir-182	1.540	1.57E-13	1.94E-12
hsa-mir-449c	1.545	0.022725218	0.035808147
hsa-mir-3187	1.548	9.69E-06	3.22E-05
hsa-mir-3651	1.580	1.77E-08	9.59E-08
hsa-mir-6744	1.586	0.004208291	0.007695422
hsa-mir-1228	1.601	7.81E-12	7.24E-11
hsa-mir-19a	1.617	3.41E-19	8.71E-18
hsa-mir-1305	1.627	0.001813868	0.003632045
hsa-mir-1910	1.639	0.001278622	0.002668016
hsa-mir-4523	1.645	7.74E-05	0.000220446
hsa-mir-3944	1.648	7.86E-08	3.85E-07

hsa-mir-3689e	1.654	0.010432754	0.017450022
hsa-mir-216b	1.675	0.001278173	0.002668016
hsa-mir-6715b	1.678	0.000270581	0.00065358
hsa-mir-501	1.681	1.31E-24	5.03E-23
hsa-mir-584	1.701	1.28E-12	1.32E-11
hsa-mir-3690-1	1.703	3.81E-07	1.65E-06
hsa-mir-7705	1.709	2.40E-12	2.32E-11
hsa-mir-335	1.710	2.20E-18	5.01E-17
hsa-mir-4517	1.715	0.000192497	0.000481529
hsa-mir-662	1.736	0.001142275	0.002413379
hsa-mir-1304	1.738	3.96E-07	1.70E-06
hsa-mir-6854	1.747	6.40E-11	5.24E-10
hsa-mir-4691	1.754	0.000391815	0.000909918
hsa-mir-96	1.765	1.99E-15	2.99E-14
hsa-mir-217	1.774	7.37E-09	4.12E-08
hsa-mir-2115	1.781	2.14E-06	8.22E-06
hsa-mir-4525	1.800	2.40E-05	7.38E-05
hsa-mir-548az	1.811	0.00028029	0.000675098
hsa-mir-506	1.831	0.003201259	0.005983729
hsa-mir-301b	1.867	8.28E-12	7.58E-11
hsa-mir-614	1.873	0.0002876	0.00068877
hsa-mir-877	1.878	1.42E-15	2.26E-14
hsa-mir-3937	1.882	0.00173421	0.003489114
hsa-mir-146b	1.886	8.28E-29	5.37E-27
hsa-mir-1246	1.886	0.000215953	0.000530753
hsa-mir-1254-2	1.890	6.10E-09	3.47E-08
hsa-mir-4777	1.892	8.18E-10	5.27E-09
hsa-mir-3176	1.895	4.92E-08	2.46E-07
hsa-mir-622	1.900	0.001200159	0.002516751
hsa-mir-3654	1.901	0.000144446	0.000373592
hsa-mir-7641-1	1.904	0.000344095	0.000805755
hsa-mir-183	1.939	1.85E-17	3.35E-16
hsa-mir-211	1.943	0.00155892	0.003189733
hsa-mir-4687	1.948	1.14E-05	3.75E-05
hsa-mir-21	1.959	1.27E-52	1.07E-49
hsa-mir-4745	1.984	1.51E-06	6.12E-06
hsa-mir-3689f	1.993	0.004397887	0.007990126
hsa-mir-551a	2.018	9.95E-08	4.71E-07
hsa-mir-1276	2.021	4.98E-06	1.75E-05
hsa-mir-374c	2.041	0.009562849	0.016155273
hsa-mir-4661	2.062	5.32E-13	6.06E-12
hsa-mir-18a	2.069	1.36E-17	2.73E-16
hsa-mir-592	2.096	4.84E-13	5.58E-12
hsa-mir-4758	2.098	8.24E-06	2.79E-05

hsa-mir-675	2.107	1.34E-06	5.46E-06
hsa-mir-3189	2.107	1.25E-08	6.86E-08
hsa-mir-3161	2.143	0.000284743	0.000683871
hsa-mir-518d	2.157	0.004648201	0.008372721
hsa-mir-5094	2.205	1.78E-06	7.11E-06
hsa-mir-4713	2.229	2.02E-06	7.90E-06
hsa-mir-4658	2.259	1.10E-06	4.50E-06
hsa-mir-6891	2.312	3.23E-07	1.43E-06
hsa-mir-615	2.369	1.77E-12	1.76E-11
hsa-mir-466	2.372	0.000514864	0.001176234
hsa-mir-7974	2.446	3.44E-10	2.36E-09
hsa-mir-937	2.552	3.23E-16	5.24E-15
hsa-mir-1283-1	2.560	0.002738305	0.005258294
hsa-mir-4664	2.620	9.08E-14	1.14E-12
hsa-mir-514a-3	2.621	4.24E-06	1.54E-05
hsa-mir-4728	2.677	2.42E-09	1.49E-08
hsa-mir-509-1	2.696	9.34E-08	4.53E-07
hsa-mir-449a	2.718	6.31E-05	0.000183332
hsa-mir-519e	2.767	0.0015435	0.003173586
hsa-mir-508	2.834	1.18E-10	8.99E-10
hsa-mir-573	2.851	1.27E-10	9.60E-10
hsa-mir-3662	2.856	3.93E-13	4.61E-12
hsa-mir-3923	2.872	0.001145748	0.002414664
hsa-mir-935	2.918	8.06E-15	1.11E-13
hsa-mir-519d	2.919	0.003074968	0.0057991
hsa-mir-934	3.004	3.48E-06	1.30E-05
hsa-mir-509-3	3.043	1.30E-09	8.12E-09
hsa-mir-514a-1	3.049	1.99E-07	9.14E-07
hsa-mir-509-2	3.072	1.45E-09	8.97E-09
hsa-mir-521-2	3.076	0.000265428	0.000642976
hsa-mir-514a-2	3.094	6.82E-07	2.85E-06
hsa-mir-524	3.108	0.003665403	0.006776173
hsa-mir-1911	3.190	0.000160088	0.000407716
hsa-mir-302c	3.215	0.000223966	0.000545675
hsa-mir-1323	3.223	0.002810504	0.005360304
hsa-mir-517b	3.240	0.002337776	0.00456191
hsa-mir-515-2	3.251	0.001107184	0.002351022
hsa-mir-135b	3.255	3.54E-27	1.86E-25
hsa-mir-517a	3.272	0.002209912	0.004342553
hsa-mir-549a	3.414	3.09E-14	4.13E-13
hsa-mir-515-1	3.586	0.000319624	0.000754741
hsa-mir-122	3.657	0.000102932	0.000280816
hsa-mir-498	3.701	0.000714857	0.001565259
hsa-mir-548f-1	3.712	9.33E-12	8.37E-11

hsa-mir-184	3.790	2.37E-08	1.26E-07
hsa-mir-516b-2	3.874	0.000129559	0.000341306
hsa-mir-1283-2	3.907	0.000144473	0.000373592
hsa-mir-302d	3.924	0.000147851	0.000378839
hsa-mir-523	4.346	3.89E-05	0.000118433
hsa-mir-519b	4.359	6.75E-05	0.000194943
hsa-mir-520d	4.365	0.000193945	0.000483715
hsa-mir-516b-1	4.395	9.67E-05	0.000268087
hsa-mir-4652	4.478	8.56E-12	7.75E-11
hsa-mir-519c	4.490	3.93E-05	0.000119032
hsa-mir-517c	4.509	0.000131898	0.000346387
hsa-mir-518e	4.557	0.00010363	0.000281807
hsa-mir-196b	4.565	4.35E-26	1.93E-24
hsa-mir-518a-2	4.630	4.95E-05	0.000146398
hsa-mir-525	4.660	0.000113156	0.000303792
hsa-mir-483	4.746	1.07E-12	1.12E-11
hsa-mir-5589	4.748	5.35E-12	5.07E-11
hsa-mir-196a-1	4.801	1.62E-33	1.52E-31
hsa-mir-516a-1	4.809	1.18E-05	3.86E-05
hsa-mir-521-1	4.821	8.73E-06	2.92E-05
hsa-mir-518b	4.827	1.31E-05	4.22E-05
hsa-mir-196a-2	4.830	1.03E-32	7.27E-31
hsa-mir-518c	4.916	4.73E-05	0.000141402
hsa-mir-526b	4.982	8.20E-06	2.79E-05
hsa-mir-520a	4.996	2.01E-05	6.25E-05
hsa-mir-518a-1	4.997	9.97E-05	0.000274715
hsa-mir-520e	5.091	1.43E-05	4.58E-05
hsa-mir-516a-2	5.202	3.80E-06	1.40E-05
hsa-mir-302a	5.406	4.53E-06	1.63E-05
hsa-mir-520g	5.420	1.70E-05	5.35E-05
hsa-mir-527	5.436	1.80E-05	5.63E-05
hsa-mir-302b	5.460	4.23E-06	1.54E-05
hsa-mir-520h	5.510	1.21E-05	3.94E-05
hsa-mir-519a-1	5.652	8.54E-08	4.16E-07
hsa-mir-520b	5.762	7.95E-06	2.74E-05
hsa-mir-512-1	5.870	2.11E-06	8.17E-06
hsa-mir-519a-2	5.884	3.64E-06	1.35E-05
hsa-mir-1269a	5.995	5.48E-10	3.67E-09
hsa-mir-522	6.060	4.29E-06	1.55E-05
hsa-mir-518f	6.078	3.28E-06	1.23E-05
hsa-mir-552	6.210	1.12E-18	2.70E-17
hsa-mir-520c	6.294	1.81E-06	7.15E-06
hsa-mir-105-1	6.347	2.41E-12	2.32E-11
hsa-mir-512-2	6.426	3.20E-07	1.43E-06

hsa-mir-105-2	6.458	6.23E-13	7.01E-12
hsa-mir-767	6.604	7.54E-13	8.36E-12
hsa-mir-1269b	6.738	4.39E-09	2.53E-08
hsa-mir-520f	6.968	1.86E-07	8.59E-07
hsa-mir-371a	7.067	2.30E-07	1.04E-06
hsa-mir-373	7.645	9.57E-08	4.61E-07
hsa-mir-372	8.063	3.24E-09	1.92E-08

Supplementary Table 2. Identification of the Prognostic miRNAs from the TCGA Cohort.

Variable	Univariable analysis		Multivariable analysis		
	HR	*P* value	HR	*P* value	coefficient
hsa-mir-708	1.216	0.002	1.139	0.061	0.13047
hsa-mir-7-3	0.858	0.003	0.855	0.007	-0.15620
hsa-mir-378i	0.734	0.006	0.690	0.001	-0.37045
hsa-mir-4793	0.554	0.006	0.549	0.008	-0.60046
hsa-mir-7-2	0.863	0.006			
hsa-mir-100	1.120	0.006			
hsa-mir-30a	1.178	0.007			
hsa-mir-137	1.154	0.011			
hsa-mir-216a	1.142	0.012			
hsa-mir-6715b	0.830	0.013			
hsa-mir-96	0.876	0.016			
hsa-mir-1292	0.822	0.017			
hsa-mir-500b	0.835	0.020			
hsa-mir-217	1.114	0.024			
hsa-mir-3161	0.820	0.025			
hsa-mir-532	0.807	0.041			
hsa-mir-466	0.762	0.042	0.792	0.089	-0.23334
hsa-mir-3144	0.785	0.042	0.774	0.039	-0.25687
hsa-mir-3923	1.161	0.046	1.294	0.007	0.25761
hsa-mir-145	1.098	0.046	0.869	0.035	-0.14044
hsa-mir-549a	0.885	0.047	0.855	0.019	-0.15682
hsa-mir-181b-1	1.187	0.047	1.259	0.014	0.22993
hsa-mir-365a	1.210	0.049	1.172	0.145	0.15853
hsa-mir-365b	1.210	0.050			
